# Advancing Health Disparities Research in Population Health

**DOI:** 10.5888/pcd15.180588

**Published:** 2018-11-29

**Authors:** Leonard Jack

**Figure:**
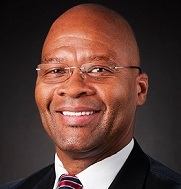
Leonard Jack Jr, PhD, MSc

The mission of *Preventing Chronic Disease* (PCD) is to promote dialogue among researchers, practitioners, and policy makers worldwide on the integration and application of research findings and practical experience to improve population health (1). Published by the Centers for Disease Control and Prevention (CDC), PCD is a peer-reviewed journal respected for its integrity and relevance to chronic disease prevention and whose articles are authored by experts worldwide. PCD is committed to publishing content that elucidates worldwide understanding of health disparities and determinants linked to disparate health outcomes. Toward that end, PCD was fortunate to have had the expertise of Dr Tim Cunningham as an associate editor. Until his untimely passing (2), Dr Cunningham provided exemplary review and oversight of manuscripts related to social determinants of health and health disparities. Through his efforts, PCD published critical research on this important topic. In honor of Dr Cunningham’s career and in appreciation for his service to the journal, PCD is dedicating to his memory this special collection of articles on effective and innovative ways to address causes of disparities from a multifactorial perspective.

Healthy People 2020 defines health disparities as “a particular type of health difference that is closely linked with social, economic, and/or environmental disadvantage” (3). As part of its mission, PCD has published papers identifying the effect of behavioral, psychological, genetic, environmental, biological, and social factors on health outcomes. PCD has also sought out research on the effectiveness of interventions addressing these factors, with the focus on reducing the disproportionate burden of chronic diseases among at-risk populations. This collection features 9 articles that address this topic from multiple perspectives:

1. The influence of implementation factors on the efficacy of school-based behavioral change interventions in low-income schools;
2. The economic factors linked to food insecurity and dietary consumption on obesity among diverse populations;
3. The relationships between consuming nuts, obesity-related foods, and body mass index among overweight and obese African American women in a rural setting;
4. The differences in health care services for diabetes care between men and women;
5. The influence of income, employment status, and education level on the prevalence of chronic disease among American Indian/Alaska Natives;
6. The contribution of falls and fall-related injuries to injury and death among older adults with chronic kidney disease;
7. The influence of sedentary behavior and the use of electronic screen devices among Mexican-origin children;
8. The creation of a diabetic retinopathy screening tool for a low-income population; and
9. The building of chronic disease epidemiology, surveillance, and evaluation in state and local health departments.

Childhood obesity continues to be a national concern, especially among low-income households (4). Blaine and colleagues described efforts to implement the “Eat Well and Keep Moving” and “Planet Health” behavioral change interventions as part of the Massachusetts Childhood Obesity Research Demonstration (MA-CORD) project in 2 school districts facing resource limitations and competing priorities (5). Researchers shared important insights on the role that key implementation outcomes such as fidelity, cost, reach, and sustainability played in school participation and sustainability of intervention activities.

The effect of short-term and long-term economic strain on the health and well-being of individuals and families is well established in published literature (6). Economic factors have been linked to food insecurity and obesity across the life stages (7). Using a spatial-based approach, Kim and colleagues identified new insights into the relationship between county-level income inequality, poverty, and obesity prevalence across New York State (8). Researchers found that higher income inequality was associated with lower obesity rates and that higher percentages of poverty were associated with higher obesity rates.

High obesity rates among African Americans continue to be a tremendous public health concern (9). High obesity rates have been linked to numerous factors, including biology, dietary consumption, population characteristics, access to care, socioeconomic status, and environment (10). Sterling and coauthors conducted research that monitored and analyzed changes in nut intake, other obesity-related foods (red or processed meats, added sugars), and body mass index during a 2-year weight loss intervention (11). The weight loss intervention targeted 383 overweight and obese African American women living in rural Alabama and Mississippi. Researchers found that nut consumers had a lower body mass index than non-nut eaters. Even after accounting for kilocalorie consumption and physical activity engagement, weight loss by the end of the intervention was significant among nut consumers but not among non-nut consumers. Researchers found that intervention results were linked to nut consumers consuming less red meat than non-nut consumers and greater amounts of other nutritionally rich foods, such as fruits and vegetables.

The existence of disparities in the use of health care services by men and women has been the subject of increased empirical study in recent years (12,13). Mesa observed 100 patients with type 2 diabetes aged 45 or older who lived in Ventura County, California, to compare differences in health care services (hemoglobin A_1c_ test, cholesterol test, and retina examination) between men and women (14). During 1 year, although men and women had access to similar health care services for diabetes, men had higher hemoglobin A_1c_ levels and lower rates of showing up for appointments. Findings from this study provide evidence that continued efforts are needed to identify motivating factors to increase appointment scheduling and attendance among men.

Chronic diseases such as heart disease, diabetes, kidney disease, and chronic lower respiratory disease disproportionately affect American Indians/Alaska Native populations, resulting in low life expectancy (15). Adamsen and colleagues conducted a national survey to measure the influence of income, employment status, and education level on the prevalence of chronic disease in a sample of 14,632 American Indians/Alaska Natives from 2011 through 2014 (16). Researchers found that most (89.7%) study participants were diagnosed with at least 1 chronic disease. American Indians/Alaska Natives with middle-to-low income levels and those who were unemployed were more likely to have received a diagnosis of a chronic disease. The authors discussed how economic development and job creation may decrease the prevalence of chronic disease in tribal communities.

Falls and fall-related injuries are the leading cause of injury and death among adults aged 65 or older (17), especially among those with chronic kidney disease (18). Kistler and colleagues performed a secondary analysis of 157,753 adults aged 65 or older in the 2014 Behavioral Risk Factor Surveillance System (19). Researchers found that adults aged 65 or older with chronic kidney disease were at increased risk of falling compared with adults in the same age range without chronic kidney disease. Researchers also found that modifiable factors such as physical function and recent exercise were most closely related to reduced risk and could be an appropriate target for fall prevention and rehabilitation programs.

Diverse factors, including family history, behavior, dietary habits, and environmental characteristics, simultaneously influence obesity among children in the United States (20). McDonald and her team of researchers examined sedentary behavior and the use of electronic screen devices among low-income Mexican-origin children aged 6 to 10 years living in rural communities near the US–Mexico border (21). Through interviews of 202 parents, researchers found that increased odds of heavy screen use were associated with having a television on while children ate. Parents reported that children also had access to electronic devices, social media, and the internet. Consistent with previously published research, this research affirmed the need to reduce screen time among children, particularly those at high risk for obesity.

Diabetes is a major public health crisis in Mexico, with mortality rates among the highest in the world (22). Diabetes is associated with complications, such as diabetic retinopathy, that impede quality of life among patients (23). Last year’s PCD Student Research Paper Contest winner in the graduate (master’s degree) category, authored by Mendoza-Herrera and colleagues, presented research results on a tool they developed to screen for diabetic retinopathy in a low-income population (24). These researchers developed the screening tool after analyzing biochemical, clinical, anthropometric, and sociodemographic information on 1,000 adults living with diabetes in low-income communities in Mexico. They developed a low-cost and easy-to-use screening tool that accounted for risk factors for diabetic retinopathy such as time since diabetes diagnosis, high blood glucose levels, systolic hypertension, and physical inactivity.

And finally in this collection, PCD examined the unique position of public health workers in state and local health departments to address social determinants of health, health inequities, and population health improvements across a range of chronic conditions in the United States (25). Calanan and colleagues described the efforts of CDC’s State Chronic Disease Epidemiology Assignee Program, a national program designed to build state and local chronic disease epidemiology, surveillance, and evaluation capacity by placing CDC field assignees in state and local health departments (26). The authors discussed how these assignees provide assistance in critical areas including conducting epidemiologic studies, building surveillance systems, evaluating chronic disease prevention and control programs, analyzing data, and training entry-level and mid-level chronic disease epidemiologists.

The articles selected for this collection demonstrate PCD’s commitment to publishing cutting-edge research for researchers, practitioners, and policy makers to better understand the multifactorial causes of health disparities, so they can develop the most effective strategies for improving health outcomes. Findings across research shared in this collection highlight the importance of employing effective interventions that address both individual and contextual factors (27). In dedicating this special collection to Dr Cunningham for his career as a social epidemiologist, published author, and esteemed PCD associate editor, we honor the excellent work that has been accomplished so far and promise to continue identifying and publishing health disparities research that increases the public health field’s understanding of what actions to take. Authors are encouraged to visit the Author’s Corner section of the journal’s website at https://www.cdc.gov/pcd/for_authors/index.htm to learn more about article types that best fit their research addressing population-based approaches to ameliorate health disparities.
